# Evaluating the Occurrence of Rare Variants in the Complement Factor H Gene in Patients With Early-Onset Drusen Maculopathy

**DOI:** 10.1001/jamaophthalmol.2021.4102

**Published:** 2021-10-14

**Authors:** Anita de Breuk, Thomas J. Heesterbeek, Bjorn Bakker, Timo Verzijden, Yara T. E. Lechanteur, Caroline C. W. Klaver, Anneke I. den Hollander, Carel B. Hoyng

**Affiliations:** 1 Donders Institute for Brain, Cognition and Behaviour, Nijmegen, the Netherlands; 2 Department of Ophthalmology, Radboud University Medical Center, Nijmegen, the Netherlands; 3 Department of Ophthalmology, Erasmus Medical Center, Rotterdam, the Netherlands; 4 Department of Epidemiology, Erasmus Medical Center, Rotterdam, the Netherlands; 5 Institute of Molecular and Clinical Ophthalmology, Basel, Switzerland

## Abstract

**Importance:**

Early-onset drusen maculopathy (EODM) is a severe disease and can lead to advanced macular degeneration early in life; however, genetic and phenotypic characteristics of individuals with EODM are not well studied.

**Objective:**

To identify genotypic and phenotypic characteristics of individuals with EODM.

**Design, Setting, and Participants:**

This case-control study collected data from the European Genetic Database from September 2004 to October 2019. A total of 89 patients with EODM diagnosed at 55 years or younger and 91 patients with age-related macular degeneration (AMD) diagnosed at 65 years or older were included.

**Exposures:**

Coding regions of *CFH*, *CFI*, *C3*, *C9*, *CFB*, *ABCA4*, *PRPH2*, *TIMP3*, and *CTNNA1* genes were sequenced, genetic risk scores (GRS) were calculated based on 52 AMD-associated variants, and phenotypic characteristics on color fundus photographs were analyzed comparing patients with EODM and AMD.

**Main Outcomes and Measures:**

GRS, frequency of rare genetic complement variants, and phenotypic characteristics.

**Results:**

This case-control study included 89 patients with EODM (mean [SD] age, 51.8 [8.7] years; 58 [65.2%] were female) and 91 patients with AMD (mean [SD] age, 77.6 [6.1] years; 45 [49.5%] female). At a mean (SD) age of 56.4 (7.3) years, 40 of 89 patients with EODM (44.9%) were affected by geographic atrophy or choroidal neovascularization. A lower GRS was observed in patients with EODM compared with patients with AMD (1.03 vs 1.60; *P* = .002), and 27 of 89 patients with EODM (30.3%) carried rare variants in the *CFH* gene compared with 7 of 91 patients with AMD (7.7%). Carriership of a rare *CFH* variant was associated with EODM (odds ratio, 7.2; 95% CI, 2.7-19.6; *P* < .001). A large macular drusen area (more than 50% covered with drusen) was observed in patients with EODM (24 of 162 eyes [14.8%]) compared with patients with AMD (9 of 164 eyes [5.5%]) (odds ratio, 4.57; 95% CI, 1.5-14.1; *P* = .008).

**Conclusions and Relevance:**

A large proportion of patients with EODM in this study carried rare *CFH* variants, with most of the identified *CFH* variants clustered in the first 7 complement control protein domains affecting factor H and factor H–like 1. Because EODM frequently leads to advanced macular degeneration at an early age and can result in many years of vision loss, this study supports targeting the complement system and sequencing the *CFH* gene in patients with EODM to improve genetic counseling and future treatments for AMD.

## Key Points

**Question:**

What are the genotypic and phenotypic characteristics of patients with early-onset drusen maculopathy?

**Findings:**

In this case-control study, patients with early-onset drusen maculopathy were frequently carriers of rare genetic variants in the complement factor H gene and were characterized by the presence of a large macular drusen area and lower genetic risk scores compared with patients with age-related macular degeneration.

**Meaning:**

Sequencing of the complement factor H gene is important in considering future treatments targeting the complement system in patients with early-onset drusen maculopathy. Furthermore, the presence of a large macular drusen area supports the severe phenotype in these patients, who may be at high risk of developing geographic atrophy or choroidal neovascularization.

## Teaser

This case-control study evaluates genotypic and phenotypic characteristics of patients with early-onset drusen maculopathy.

## Introduction

The advanced stages of age-related macular degeneration (AMD), geographic atrophy (GA), and choroidal neovascularization (CNV) can lead to severe vision loss. The prevalence of advanced AMD rises from 0.5% in individuals aged 65 to 69 years to 9.8% in those 85 years or older.^1^ Both genetic and nongenetic risk factors are implicated in the disease pathogenesis. The first major discoveries included the association of variants in complement factor H (*CFH*) and age-related maculopathy susceptibility 2 (*ARMS2*) genes with AMD. Subsequently, more than 52 genetic variants at 34 genomic loci were found to be associated with AMD, most of which are common variants.^2^ In addition, rare variants in *CFH*, complement factor I (*CFI*), complement 3 (*C3*), complement 9 (*C9*), tissue inhibitor of metalloproteinases (*TIMP3*), and solute carrier family 16 member 8 (*SLC16A8*) genes have been identified in AMD.^2,3,4,5,6,7,8,9^

Although AMD is considered a late-onset macular disease, some individuals may develop drusen and other disease characteristics associated with AMD at a much younger age. Patients with early-onset drusen maculopathy (EODM) may develop vision-threatening advanced disease stages earlier in life. A variety of terms have been used to describe drusen at a young age, such as *early-onset macular drusen* and *early-onset AMD*.^7,10,11,12,13^ It remains unclear whether EODM is an early manifestation of AMD or a separate disease entity. Given the early age of onset, a genetic component might be considered. Three studies have identified rare *CFH* variants in families with cuticular drusen and in families with early-onset macular drusen.^13,14,15^ To our knowledge, other complement-associated genes have not been associated with EODM so far; however, a comprehensive genetic analysis is lacking. Furthermore, specific inherited macular dystrophies (eg, late-onset Stargardt disease, central areolar choroidal dystrophy [CACD], and Sorsby fundus dystrophy [SFD]) can present with drusen or other characteristics associated with AMD, such as GA or CNV, which may resemble EODM.^16,17^

In this study, we aimed to describe phenotypic characteristics, determine the contribution of 52 AMD-associated variants using genetic risk score (GRS), and perform a comprehensive analysis of the coding and splice-site regions of *CFH*, *CFI*, *C3*, *C9*, *CFB*, *ABCA4*, *PRPH2*, *TIMP3*, and *CTNNA1* in a cohort of 89 patients with EODM and compare these characteristics with characteristics of patients with AMD.

## Methods

### Study Population

We collected data on patients diagnosed with EODM from the European Genetic Database (EUGENDA). Patient recruitment took place from September 2004 to October 2019. This study was approved by the medical ethics committee of the Radboud University Medical Center, Nijmegen, the Netherlands, and all study participants provided written informed consent. No compensation or incentive was offered to patients for participation in this study. This research adhered to the tenets of the Declaration of Helsinki.

EODM was defined as any sign of age-related maculopathy (eTable 1 in the Supplement) diagnosed at 55 years or younger. This age threshold was based on exclusion criteria of the MACUSTAR clinical study,^18^ which applied an age threshold of 55 years for patients with AMD. Twenty-four patients were diagnosed with EODM from ages 56 to 65 years. Retinal images were evaluated by a clinical expert (C.B.H.) and patients were assigned to the EODM group, as they presented with a severe EODM phenotype at an early age, which was the case for 20 of 24 patients. The total group consisted of 89 patients with EODM. To compare the genotypic and phenotypic characteristics of patients with EODM, we selected a group of patients with AMD diagnosed at 65 years or older from EUGENDA, for whom detailed grading was available. All individuals were unrelated and from European descent.

### Image Assessment and Grading

Topcon TRC 50IX (Topcon Corporation), Topcon DRI Triton (Topcon Corporation), and Spectralis HRA+OCT (Heidelberg Engineering) cameras were used to capture color fundus photographs and optical coherence tomography images. Grading was performed by experienced graders under the supervision of a senior retinal specialist (C.C.W.K.) according to the international classification system^19^ based on the Wisconsin Age-Related Maculopathy Grading System^20^ and reclassified to the Rotterdam classification (eTable 1 in the Supplement).^21^ The following AMD-related features were graded: predominant drusen type; largest drusen size and drusen area in grid; presence of small, intermediate, and large drusen; extramacular drusen (EMD); increased pigment; RPE degeneration; presence of GA or CNV; and, if a CNV was present, serous detachment, subretinal hemorrhage, fibrous scar, and hard exudates.

### Genotyping

Genotyping was performed as described previously by whole-exome sequencing,^22^ single-molecule molecular inversion probes (smMIPs) in combination with next-generation sequencing^23^ or exome chip analysis.^2^ For a small number of patients without whole-exome sequencing or smMIPs data, a panel of complement genes was sequenced (*CFH*, *CFI*, *C3*, and *CFB*) in clinical diagnostic setting (Radboud University Medical Center) using Sanger sequencing or next-generation sequencing (Illumina NextSeq 500) after enrichment with smMIPs. All genotyping data passed quality control, as described previously.^2,22,23^ In whole-exome sequencing and smMIPs data, we evaluated coding and splice-site regions of *CFH*, *CFI*, *C3*, *C9*, and *CFB* to identify carriers of rare variants (minor allele frequency less than 0.01, based on the non-Finnish European population in the Genome Aggregation Database). In addition, we filtered for carriers of 2 or more rare or low-frequency protein-altering or splice-site variants in *ABCA4* (autosomal recessive late-onset Stargardt disease), carriers of *PRPH2* Arg142Trp (autosomal dominant CACD), and carriers of rare protein-altering or splice-site variants in *TIMP3* and *CTNNA1* (SFD and butterfly-shaped pigment dystrophy, respectively) to identify patients with inherited macular dystrophies resembling EODM.^16,17,24^ Other genes associated with macular dystrophies mimicking EODM were not included because smMIPs genotyping contained only the before-mentioned 4 macular dystrophy genes. Exome chip and smMIPs data were used to calculate a GRS based on 52 AMD-associated variants,^2^ as described previously.^23^ Further details are depicted in eTable 2 in the Supplement.

### Statistical Analysis

General characteristics were compared using Mann-Whitney *U* or χ^2^ tests and are presented as medians with corresponding IQRs or numbers with corresponding percentages. We performed binary logistic regression analysis to compare carriership of rare variants between patients with EODM and those with AMD, adjusting for sex and smoking. Mann-Whitney *U* tests and Spearman ρ correlation coefficient were used to analyze GRS between phenotype and disease stage. Mixed model analysis using univariable binary or multinomial logistic regression analysis, correcting for intereye correlation, was used to compare phenotypic characteristics between both groups. Sex, smoking, and carriership of a rare *CFH* variant were included as covariates. Results are presented as odds ratios (ORs) with corresponding 95% CIs. All *P* values were 2-sided, and there were no adjustments to *P* values for multiple analyses. Significance for *P* was set at .05. All analyses were performed using SPSS version 22 (IBM Corp).

## Results

### Description of the EODM Cohort

**Figure 1.**
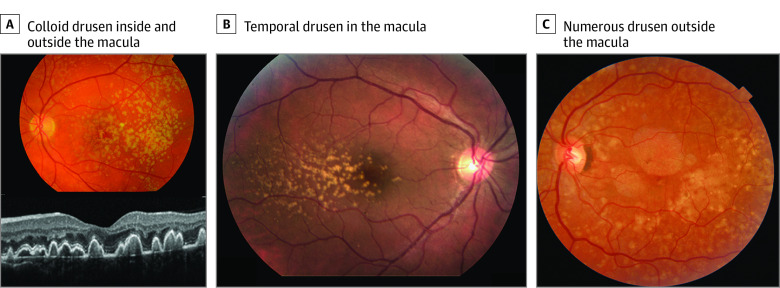
Patients With Early-Onset Drusen Maculopathy With Large Colloid Drusen, Temporal Drusen, and Extramacular Drusen A, Color fundus photograph and optical coherence tomography of a female individual aged 35 years with large colloid drusen located inside and outside the macula. This patient is a carrier of rare variants in the complement factor H (*CFH*) gene (Gln950His) and in the complement 3 (*C3*) gene (Arg735Trp) (genetic risk score [GRS], 0.23). B, Color fundus photograph of a female individual aged 37 years with predominantly temporal drusen in the macula (GRS, −1.37). C, Color fundus photograph of a female individual aged 50 years with numerous drusen outside the macula (GRS, −0.43).

The EODM cohort consisted of individuals with a heterogenous spectrum of drusen phenotypes, including 7 individuals with extensive EMD, 6 individuals with large colloid drusen, 3 individuals with temporal drusen, and multiple individuals with cuticular drusen (Figure 1; eFigure 1 in the Supplement). The median (IQR) age at diagnosis in the EODM cohort was 49.0 (45.0 to 55.0) years, 58 individuals (65.2%) were female, and 40 of 89 (44.9%) were affected by advanced macular degeneration in at least 1 eye at a mean (SD) age of 56.4 (7.3) years. Within the advanced group of 40 individuals, 16 (40.0%) had GA, 18 (45.0%) had CNV, and 6 (15.0%) had a combination of GA and CNV (Table 1). Six individuals reported another relevant medical condition, including 3 individuals with *C3* glomerulopathy (eTable 3 in the Supplement).

**Table 1.** General Characteristics of Early-Onset Drusen Maculopathy (EODM) and Age-Related Macular Degeneration (AMD) Cohorts

Characteristic	No. (%)	
	EODM (n = 89)	AMD (n = 91)
Sex		
Female	58 (65.2)	45 (49.5)
Male	31 (34.8)	46 (50.5)
Age, mean (SD), y	51.8 (8.7)	77.6 (6.1)
Age at diagnosis, median (IQR), y	49.0 (45.0-55.0)	75.0 (71.0-80.0)
Smoking status^a^		
Never	35 (39.3)	21 (23.1)
Previous use	31 (34.8)	47 (51.6)
Current use	15 (16.9)	14 (15.4)
AMD stage^b^		
1	2 (2.2)	6 (6.6)
2	20 (22.5)	5 (5.5)
3	27 (30.3)	5 (5.5)
4		
GA	16 (18.0)	29 (31.9)
CNV	18 (20.2)	28 (30.8)
Combination (CNV and GA)	6 (6.7)	18 (19.8)
GRS, median (IQR)	1.03 (0.16-1.94)	1.60 (0.84-2.64)
MAF, No. of minor alleles/total No. of alleles (%)		
*CFH* rs570618	74/148 (50.0)	97/174 (55.7)
*ARMS2* rs3750846	34/148 (23.0)	67/174 (38.5)

Abbreviations: *ARMS2*, age-related maculopathy susceptibility 2; *CFH*, complement factor H; CNV, choroidal neovascularization; GA, geographic atrophy; GRS, genetic risk score; MAF, minor allele frequency.

^a^ Percentages do not add up to 100 because smoking status was not available for 17 individuals.

^b^ AMD stage according to Rotterdam classification.

### Analysis of 52 Single-Nucleotide Variations in Individuals With EODM

The median (IQR) GRS in individuals with EODM (1.03 [0.16 to 1.94]) was lower compared with that of individuals with AMD (1.60 [0.84 to 2.64]) (*P* = .002), and there was a positive correlation between disease stage and GRS (ρ, 197; *P* = .01). Next, we stratified patients into early disease stage (Rotterdam classification, 1-3) and late disease stage (Rotterdam classification, 4). We observed a slightly lower GRS in patients with EODM compared with patients with AMD in early disease stages (0.83 vs 1.42; *P* = .22) and a lower GRS in those in late disease stages (1.34 vs 1.67; *P* = .04) (eFigure 2 in the Supplement). The median (IQR) GRSs in patients with EODM with extensive EMD and temporal drusen were low (−0.27 [−0.35 to −0.02] and −0.07 [−0.72 to 0.43], respectively) compared with the GRS in individuals with large colloid drusen (1.64 [0.32 to 2.65]).

The minor allele frequencies of main risk alleles *CFH* rs570618 and *ARMS2/HTRA1* rs3750846 were lower in individuals with EODM compared with individuals with AMD (74 of 148 [50.0%] vs 97 of 174 [55.7%]; χ^2^ *P* = .30 and 34 of 148 [23.0%] vs 67 of 174 [38.5%]; χ^2^ *P* = .003, respectively).

### Analysis of Rare Variants in Complement Genes

**Figure 2.**
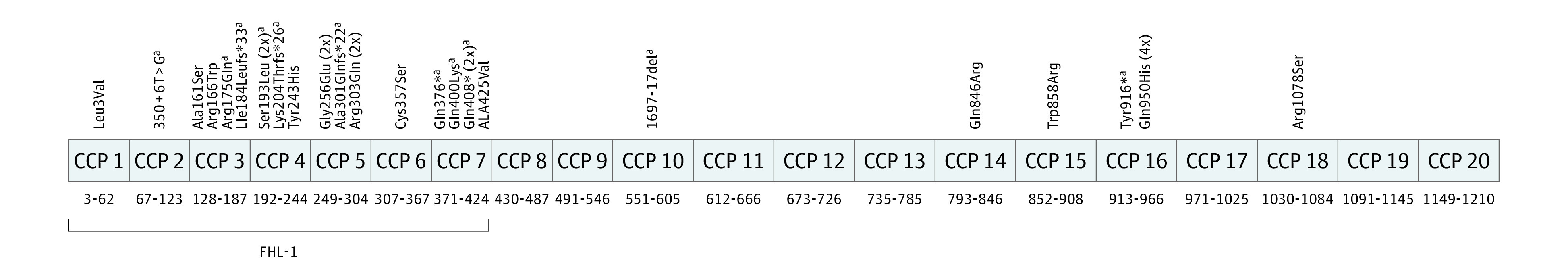
Distribution of Rare Complement Factor H Variants in Patients With Early-Onset Drusen Maculopathy Numbers under the complement control protein (CCP) domains represent the amino acid positions. FHL-1 indicates factor H–like 1.^25^ ^a^Functional variants.

Thirty-five of 89 patients with EODM (39.3%) carried 1 or more rare variant in one of the complement genes. Of these patients, 80.0% carried 1 rare complement variant, 14.3% carried 2, and 5.7% carried 3 (eTable 4 in the Supplement). Most were identified in *CFH*. In total, 27 of 89 patients with EODM (30.3%) carried a rare *CFH* variant compared with 7 of 91 patients with AMD (7.7%). Carriership of a rare *CFH* variant was associated with EODM, adjusted for sex and smoking (OR, 7.2; 95% CI, 2.7-19.6; *P* < .001) (eTable 5 in the Supplement). Smoking status was incomplete for 17 individuals. Characteristics of individuals with complete and incomplete smoking data are depicted in eTable 6 in the Supplement. Most of the rare unique *CFH* variants (17 of 23 [73.9%]) affect the first 7 complement control protein (CCP) domains (part of the factor H [FH] and factor H–like 1 [FHL-1; a shorter isoform of FH resulting from alternative splicing of *CFH*] proteins). The clustering of rare *CFH* variants became even more prominent when selecting only variants affecting FH function: 9 of 11 unique functional *CFH* variants (81.8%) were clustered in CCPs 1 through 7 (Figure 2).^25^ We also identified 5 *C3*, 6 *C9*, 1 *CFB*, and 2 *CFI* rare variants in patients with EODM (eTables 4 and 7 in the Supplement).

### Comparison of Phenotypic Characteristics of Patients With EODM and Patients With AMD

**Figure 3.**
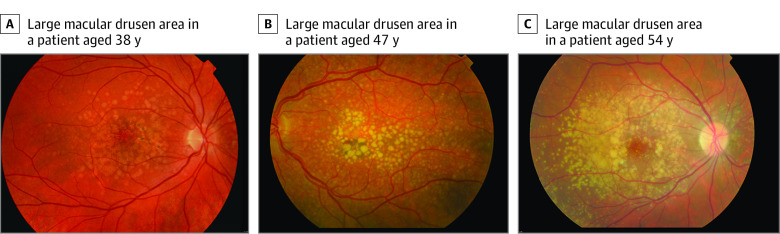
Color Fundus Photographs of 3 Patients With Early-Onset Drusen Maculopathy Showing a Large Macular Drusen Area

We compared phenotypic characteristics on color fundus photographs in 162 eyes of 81 patients with EODM and 164 eyes of 82 patients with AMD. Thirty-four eyes of 17 individuals were excluded from analysis owing to incomplete smoking data (eTable 6 in the Supplement). Four features in Table 2 (ie, proportion of grid area covered by drusen, RPE degeneration, GA, and CNV) were suggested to be different, although when using Bonferroni correction for multiple analyses, the *P* value was less than .003, wherein less than .003 (α = .05/15 tests) might be considered statistically significant. EODM was associated with a large drusen area: in 94 of 162 eyes (58.0%) of patients with EODM, 10% to 50% of the grid was covered by drusen compared with 66 of 164 eyes (40.2%) of patients with AMD (OR, 2.29; 95% CI, 1.06-4.93; *P* = .03), and in 24 of 162 eyes (14.8%) of patients with EODM, more than 50% of the grid was covered by drusen compared with 9 of 164 eyes (5.5%) of patients with AMD (OR, 4.57; 95% CI, 1.48-14.09; *P* = .008) (Figure 3). Patients with EODM tended to have larger drusen compared with patients with AMD (OR, 4.45; 95% CI, 0.40-50.11; *P* = .23 for drusen size 125μm; OR, 5.10; 95% CI, 0.51-51.62; *P* = .17 for drusen size 250 μm; and OR, 9.02; 95% CI, 0.85-95.25; *P* = .07 for drusen size greater than 250μm). Furthermore, EODM was associated with RPE degeneration within the central grid (OR, 2.51; 95% CI, 1.08-5.80; *P* = .03). When comparing advanced AMD stages between both groups, we observed a lower frequency of GA (35 of 162 eyes [21.6%] vs 66 of 164 eyes [40.2%]; OR, 0.33; 95% CI, 0.15-0.71; *P* = .005) and CNV (35 of 162 eyes [21.6%] vs 56 of 164 eyes in patients with EODM [34.1%]; OR, 0.46; 95% CI, 0.22-0.99; *P* = .046).

**Table 2.** Phenotypic Characteristics of Patients with Early-Onset Drusen Maculopathy (EODM) and Age-Related Macular Degeneration (AMD)

Characteristic	Eyes, No. (%)^a^		Odds ratio (95% CI)^b^	*P* value
	EODM (n = 162)	AMD (n = 164)		
Predominant drusen type in grid				
Hard drusen	1 (0.6)	4 (2.4)	1 [Reference]	NA
Soft drusen<C1	10 (6.2)	22 (13.4)	1.79 (0.13-23.78)	.66
Distinct drusen	5 (3.1)	28 (17.1)	0.69 (0.05-9.71)	.78
Indistinct drusen	141 (87.0)	64 (39.0)	9.40 (0.79-112.56)	.08
Reticular drusen	3 (1.9)	22 (13.4)	0.41 (0.02-7.11)	.54
Largest drusen size within grid				
<C0, 63 μm	1 (0.6)	5 (3.0)	1 [Reference]	NA
<C1, 125 μm	23 (14.2)	22 (13.4)	4.45 (0.40-50.11)	.23
<C2, 250 μm	42 (25.9)	41 (25.0)	5.10 (0.51-51.62)	.17
≥C2, 250 μm	90 (55.6)	49 (29.9)	9.02 (0.85-95.25)	.07
Reticular	4 (2.5)	21 (12.8)	0.74 (0.05-10.82)	.82
Small drusen in grid				
Absent	52 (32.1)	50 (30.5)	1 [Reference]	NA
Present	107 (66.0)	90 (54.9)	1.07 (0.53-2.14)	.85
Intermediate drusen in grid				
Absent	9 (5.6)	13 (7.9)	1 [Reference]	NA
Present	150 (92.6)	127 (77.4)	1.48 (0.46-4.80)	.51
Large drusen in grid				
Absent	24 (14.8)	32 (19.5)	1 [Reference]	NA
Present	136 (84.0)	108 (65.9)	1.77 (0.71-4.42)	.22
Proportion of grid area covered by drusen				
0%-10%	42 (25.9)	66 (40.2)	1 [Reference]	NA
10%-50%	94 (58.0)	66 (40.2)	2.29 (1.06-4.93)	.03
>50%	24 (14.8)	9 (5.5)	4.57 (1.48-14.09)	.008
Drusen outside grid				
Absent	22 (13.6)	41 (25.0)	1 [Reference]	NA
Present	138 (85.2)	122 (74.4)	1.46 (0.59-3.62)	.41
Increased pigment				
Absent	69 (42.6)	67 (40.9)	1 [Reference]	NA
<C2, 250 μm	24 (14.8)	20 (12.2)	1.23 (0.54-2.77)	.62
≥C2, 250 μm	67 (41.4)	75 (45.7)	0.91 (0.43-1.91)	.80
RPE degeneration				
Absent	101 (62.3)	130 (79.3)	1 [Reference]	NA
<C2, 250 μm	14 (8.6)	8 (4.9)	2.47 (0.82-7.42)	.11
<Central grid	32 (19.8)	21 (12.8)	2.51 (1.08-5.80)	.03
≥Central grid	3 (1.9)	3 (1.8)	1.15 (0.13-10.50)	.90
Geographic atrophy				
Absent	126 (77.8)	97 (59.1)	1 [Reference]	NA
Present	35 (21.6)	66 (40.2)	0.33 (0.15-0.71)	.005
Choroidal neovascularization				
Absent	122 (75.3)	107 (65.2)	1 [Reference]	NA
Present	35 (21.6)	56 (34.1)	0.46 (0.22-0.99)	.046
Serous detachment (if CNV)				
Absent	11 (31.4)	16 (28.6)	1 [Reference]	NA
Present	21 (60.0)	39 (69.6)	0.70 (0.16-2.99)	.62
Subretinal hemorrhage (if CNV)				
Absent	31 (88.6)	40 (71.4)	1 [Reference]	NA
Present	2 (5.7)	14 (25.0)	0.07 (0.003-1.52)	.09
Fibrous scar (if CNV)				
Absent	9 (25.7)	9 (16.1)	1 [Reference]	NA
Present	25 (71.4)	46 (82.1)	0.51 (0.07-3.78)	.50
Hard exudates (if CNV)				
Absent	29 (82.9)	35 (62.5)	1 [Reference]	NA
Present	4 (11.4)	21 (37.5)	0.11 (0.01-1.11)	.06

Abbreviations: CFH, complement factor H; CNV, choroidal neovascularization; NA, not applicable.

^a^ Percentages do not always add to 100%, because in some eyes specific features were not applicable or could not be graded.

^b^ Presented odds ratios result from univariable mixed model logistic regression analyses corrected for sex, smoking, and carriership of a rare *CFH* variant.

### Analysis of Rare Variants in *ABCA4*, *PRPH2*, *CTNNA1*, and *TIMP3*

In 1 of 89 patients with EODM (1.1%), we identified 2 or more *ABCA4* variants (Pro1948Leu and Arg212His), both classified as benign,^26^ and in 3 of 89 patients (3.4%), 1 heterozygous, likely pathogenic *ABCA4* variant was identified (Ala1038Val [2x], Arg2030Gln). No second *ABCA4* variant was found in the coding or splice-site regions. The retinal images were closely evaluated by a retinal expert (C.B.H.) and were not suspect for late-onset Stargardt disease. None of the patients with EODM carried rare protein-altering or splice-site variants in *CTNNA1* and *TIMP3*.

One individual carried the *PRPH2* Arg142Trp variant heterozygously (confirmed by Sanger sequencing). On optical coherence tomography, multiple elevations of the RPE were visible underneath the fovea. Color fundus photographs showed several small yellow deposits in the macula. Based on phenotype and supported by the high prevalence of AMD in the family, this patient was primarily diagnosed with EODM. However, reevaluation of the phenotype in combination with the *PRHP2* Arg142Trp mutation revealed that this patient was most likely affected by CACD rather than EODM (eFigure 3 in the Supplement).

## Discussion

In this case-control study, we collected data on a large and unique cohort of patients with EODM and compared genotypic and phenotypic characteristics between patients with EODM and those with AMD. A large proportion of patients with EODM presented with vision-threatening advanced macular degeneration at an early age. Despite the large heterogenicity in phenotype, we observed that EODM was associated with a large macular drusen area. Furthermore, one-third of patients with EODM carried rare *CFH* variants, suggesting that rare variants in this gene are frequently associated with EODM, which may have important implications for genetic counseling and treatment strategies.

In the literature, different terms have been used to study patients with drusen at young age. In this study, we used the term *EODM*. Although a large proportion of the patients with drusen at young age had already developed GA and/or CNV, we specifically selected patients based on the presence of drusen and therefore did not use the term *early-onset macular degeneration*. Considering the high degree of overlap in phenotypic characteristics between patients with EODM and those with AMD, EODM seems to be an early manifestation of AMD rather than a separate disease entity. Rare variants with large effect sizes could contribute to an earlier disease onset. However, rare variants were not identified in all patients with EODM, suggesting that rare variants in other genes or other nongenetic modifiers are involved in the development of EODM.

The large proportion of patients with EODM (44.9%) affected by advanced macular degeneration at a mean early age of 56.4 years is remarkable and is in stark contrast with the prevalence of advanced AMD in the general European population: 0.5% in individuals aged 65 to 69 years and 9.8% in individuals 85 years and older.^1^ The early onset of GA or CNV can have a huge impact on quality of life, as patients with EODM will likely need to cope with the results of many more years of visual impairment, in some cases already beginning during working life.

Distribution of GRS among the disease stages in our study is in line with previous studies.^23,27^ The lower GRS observed in patients with EODM could imply that EODM is associated to a lesser extent with common AMD single-nucleotide variations. In particular, this seems to be the case for patients with EODM with temporal drusen and extensive EMD who presented with a low GRS, suggesting that these drusen subtypes might be associated with different genetic factors than classic AMD. As these subgroups are small, further research should confirm this finding.

A total of 30.3% of patients with EODM carried rare *CFH* variants, considerably more than patients in the AMD group. Important to note is that a large proportion of data on patients with EODM were collected from the outpatient clinic of the Department of Ophthalmology (Radboud University Medical Center, Tertiary Referral Center), and diagnostic genetic testing (including sequencing of complement genes) is available as part of clinical care. In general, patients from families with a dense history of AMD and patients with severe disease at an early age were tested because those patients were expected to have a higher chance of carrying rare variants in complement genes.^6,7,13,14,15,28,29^ Nevertheless, the high percentage of patients with EODM carrying rare *CFH* variants is remarkable in comparison with the percentage (7.7%) of patients in the AMD group and with patients with EODM carrying rare variants in *C3* (5.6%), *C9* (6.7%), *CFB* (1.1%), and *CFI* (2.2%).

The clustering of rare *CFH* variants in CCP 1 to 7 of FH in our study is in line with a study by Taylor et al^13^ that examined 10 individuals with early-onset macular drusen from 7 families. All 6 variants were clustered in CCP domains 2 to 7 and were different from the rare *CFH* variants in our study. In 2 other studies, enrichment of rare *CFH* variants was observed in CCP domains 1 to 4 and 19 to 20 in individuals with advanced AMD,^30^ and in CCP 3, 5, and 7 in individuals with AMD.^31^ The first 7 CCP domains of FH are identical to FHL-1. Previous work by Clark et al^32^ demonstrated that FHL-1 can passively diffuse through the Bruch membrane, in contrast to full-length FH, which is unable to pass through the Bruch membrane. Clark et al suggested that FHL-1 is the main regulator that protects the Bruch membrane against the damaging effects of complement activation. Considering the protective function of FHL-1 in complement regulation, we believe that rare *CFH* variants in CCP 1 to 7 make a substantial contribution to the development of AMD and EODM.

In our study, we found that a large macular drusen area was associated with EODM. In several studies, drusen area and volume were associated with increased risk of progression to advanced AMD.^33,34^ The observation of a large macular drusen area in our study supported the severe phenotype in patients with EODM, as these patients were at risk of progression to advanced macular degeneration. In a study by Kersten et al,^28^ a large macular drusen area was associated with AMD in patients carrying rare *CFH* variants. Evaluation of the patients in our study with a drusen area of more than 50% revealed that only 5 of 27 patients with EODM and 0 of 13 patients with AMD were carrying rare *CFH* variants. Phenotypic characteristics in our study did not pass Bonferroni correction for multiple hypothesis testing; therefore, replication in a larger and/or independent cohort is recommended.

Caution is warranted, considering the phenotypic overlap between EODM and macular dystrophies, such as Mallatia Leventinese, SFD, and CACD.^16,24^ A previous family-based study by Klevering et al^35^ showed a range of phenotypes in patients with CACD carrying the *PRPH2* Arg142Trp mutation. Some patients presented with CACD without any noticeable drusen, but more importantly, in multiple patients with CACD, drusen were observed. This was also the case in 1 patient in this current study (eFigure 3 in the Supplement). Another macular dystrophy mimicking EODM is SFD, which is characterized by the formation of a CNV with or without drusen.^36^ None of the patients with EODM carried rare variants in this gene. Although we were not able to analyze all genes associated with macular dystrophies that could mimic EODM, we did not find suspicion of other macular dystrophies based on retinal imaging in the EODM cohort.

### Limitations

This study had limitations. We focused on rare variants in complement genes only; however, rare variants in genes involved in other biological pathways might also play a role in the development of EODM. Therefore, future studies could benefit from a whole genome approach, especially in individuals with a low GRS based on the 52 AMD-associated single-nucleotide variations who do not carry any rare variants in genes of the complement pathway. Furthermore, an overlap in phenotypic characteristics was observed between EODM and AMD, and our sample size might be insufficient to detect clear differences in phenotypic features. Enlarging the EODM cohort would improve the power to detect associations of phenotypic characteristics with EODM.

## Conclusions

Rare variants in *CFH* are a frequent finding in patients with EODM. Moreover, a large proportion of patients with EODM are affected by advanced macular degeneration at an early age. Our findings support targeting the complement system and sequencing the *CFH* gene in EODM for genetic counseling purposes and upcoming treatments for AMD supplementing FH protein, such as GEM103 (NCT04246866).

## Notes

Supplement. **eTable 1.** Age-related macular degeneration stages according to Rotterdam classification **eTable 2.** Fifty-two AMD-associated variants included in GRS calculation **eTable 3.** Relevant medical conditions EODM group **eTable 4.** Patients with EODM carrying rare complement variants **eTable 5.** Logistic regression analysis for the association of *CFH* rare variant carriership with EODM phenotype **eTable 6.** Characteristics complete and incomplete data **eTable 7.** Details of rare complement variants identified in patients with early-onset drusen maculopathy **eFigure 1.** Patients with EODM with large colloid drusen and temporal drusen **eFigure 2.** Genetic risk score distribution in EODM and AMD cohorts **eFigure 3.** Patient with *PRPH2* Arg142Trp mutation
eReferences
