# Supplementary material for: Evaluating the Occurrence of Rare Variants in the Complement Factor H Gene in Patients With Early-Onset Drusen Maculopathy
Source: JAMA Ophthalmol. 2021 Oct 14;139(11):1218–26. doi: 10.1001/jamaophthalmol.2021.4102 (PMC8517879; doi:10.1001/jamaophthalmol.2021.4102)
Supplement: Supplement. — eTable 1. Age-related macular degeneration stages according to Rotterdam classification eTable 2. Fifty-two AMD-associated variants included in GRS calculation eTable 3. Relevant medical conditions EODM group eTable 4. Patients with EODM carrying rare complement variants eTable 5. Logistic regression analysis for the association of CFH rare variant carriership with EODM phenotype eTable 6. Characteristics complete and incomplete data eTable 7. Details of rare complement variants identified in patients with early-onset drusen maculopathy eFigure 1. Patients with EODM with large colloid drusen and temporal drusen eFigure 2. Genetic risk score distribution in EODM and AMD cohorts eFigure 3. Patient with PRPH2 Arg142Trp mutation eReferences [file jamaophthalmol-e214102-s001.pdf]

Supplemental Online Content

de Breuk A, Heesterbeek TJ, Bakker B, et al. Evaluating the occurrence of rare variants in the complement factor H gene in patients with early-onset drusen maculopathy. *JAMA Ophthalmol*. Published online October 14, 2021. doi:10.1001/jamaophthalmol.2021.4102

eTable 1. Age-related macular degeneration stages according to Rotterdam classification

eTable 2. Fifty-two AMD-associated variants included in GRS calculation

eTable 3. Relevant medical conditions EODM group

eTable 4. Patients with EODM carrying rare complement variants

eTable 5. Logistic regression analysis for the association of *CFH* rare variant carriership with EODM phenotype

eTable 6. Characteristics complete and incomplete data

eTable 7. Details of rare complement variants identified in patients with early-onset drusen maculopathy

eFigure 1. Patients with EODM with large colloid drusen and temporal drusen

eFigure 2. Genetic risk score distribution in EODM and AMD cohorts

eFigure 3. Patient with *PRPH2* Arg142Trp mutation

eReferences

This supplemental material has been provided by the authors to give readers additional information about their work.

© 2021 de Breuk A et al. *JAMA Ophthalmology.*

eTable 1. Age-related macular degeneration stages according to Rotterdam classification

Grades Rotterdam classification	Definition
0	No signs of AMD or hard drusen (< 63 µm) only
1	Soft indistinct drusen (≥ 63 µm) or pigmentary abnormalities
2	Soft indistinct drusen (≥ 125 µm) or reticular drusen or soft indistinct drusen (≥ 63 µm) with pigmentary abnormalities
3	Soft indistinct drusen (≥ 125 µm) or reticular drusen with pigmentary abnormalities
4	Atrophic, neovascular or mixed AMD

Table detailing the different stages of age-related macular degeneration according to Rotterdam classification. AMD = age-related macular degeneration.

© 2021 de Breuk A et al. *JAMA Ophthalmology.*

eTable 2. Fifty-two AMD-associated variants included in GRS calculation

Locus name	Variant	Alternative variant
ABCA1	rs2740488	
ACAD10/BRAP	rs61941274	rs61941272 (R^2 1.0)
ADAMTS9-AS2	rs62247658	
APOE	rs429358	
APOE(EXOC3L2/MARK4)	rs73036519	
ARHGAP21	rs12357257	
ARMS2/HTRA1	rs3750846	
B3GALTL	rs9564692	
C2/CFB/SKIV2L	rs116503776/rs429608	
C2/CFB/SKIV2L	rs144629244/rs2746394	
C2/CFB/SKIV2L	rs181705462	rs114212545 (R^2 0.84)
C2/CFB/SKIV2L (PBX2)	rs114254831/rs204993	
C20orf85	rs201459901	rs117420707 (R^2 0.99)
C3	rs2230199	
C3	rs147859257	
C3 (NRTN/FUT6)	rs12019136	rs17855739 (R^2 0.95)
C9	rs62358361	
CETP	rs5817082	
CETP	rs17231506	
CFH	rs10922109	
CFH	rs570618	
CFH	rs121913059	
CFH	rs148553336	
CFH	rs187328863	rs79436252 (R^2 1.0)
CFH	rs35292876	
CFH	rs191281603	
CFH (CFHR3/CFHR1)	rs61818925	rs61818924 (R^2 0.80)
CFI	rs10033900	
CFI	rs141853578	
CNN2	rs67538026	rs113772652 (R^2 0.996)
COL4A3	rs11884770	
COL8A1	rs140647181	
COL8A1	rs55975637	
CTRB2/CTRB1	rs72802342	rs55993634 (R^2 0.89)
KMT2E/SRPK2	rs1142	
LIPC	rs2043085	
LIPC	rs2070895	
MIR6130/RORB	rs10781182	
MMP9	rs142450006	
NPLOC4/TSPAN10	rs6565597	
PILRB/PILRA	rs7803454	
PRLR/SPEF2	rs114092250	rs74767144 (R^2 0.77)
RAD51B	rs61985136	
RAD51B	rs2842339	
RDH5/CD63	rs3138141	
SLC16A8	rs8135665	
SYN3/TIMP3	rs5754227	
TGFBR1	rs1626340	
TMEM97/VTN	rs11080055	
TNFRSF10A	rs79037040/rs13278062	
TRPM3	rs71507014	
VEGFA	rs943080	

Table detailing the genetic variants included for calculation of the genetic risk score. AMD = age-related macular degeneration, GRS = genetic risk score

© 2021 de Breuk A et al. *JAMA Ophthalmology.*

eTable 3. Relevant medical conditions EODM group

Medical condition	No. of patients affected	Comments
C3 glomerulopathy (dense deposits disease [DDD] / membranoproliferative glomerulonephritis type 2 [MPGN type 2])	3	- Macular drusen can be observed in patients with C3 glomerulopathy.[1,2]<br>- Mutations in the complement factor H (*CFH*) gene are identified in patients with C3 glomerulopathy, as well as several single nucleotide polymorphisms (SNPs) in the *CFH* gene.[1,3-5]
Membranoproliferative glomerulonephritis type 1 (MPGN type 1)	1	- In contrast to C3 glomerulopathy (complement-related), MPGN type 1 (immune complex related) is not associated with drusen. However, drusen are reported in 1 case report (patient with MPGN type 1).[6]
Behcet's Disease	1	- Ocular conditions associated with Behcet's Disease include iritis, uveitis, retinal vasculitis, vitritis and optic neuropathy. Drusen are not associated with Behcet's Disease.
X-linked agammaglobulinemia (XLA)	1	- XLA is characterized by a lack of antibodies, resulting in increased risk of infections. In literature, drusen are not associated with XLA.

Table detailing medical conditions reported in early-onset drusen maculopathy patients that are relevant in light of this study.

© 2021 de Breuk A et al. *JAMA Ophthalmology.*

eTable 4. Patients with EODM carrying rare complement variants

No.	Variant 1	Source variant 1	Variant 2	Source variant 2	Variant 3	Source variant 3
1	*C9* Pro167Ser	WES				
2	*CFH* c.350+6T>G	Sanger				
3	*CFH* Gln408*	WES, smMIPs	*C3* Arg735Trp	smMIPs, exome chip, KASPar		
4	*CFH* Gln408*	Sanger				
5	*CFH* Ala301Glnfs*22	smMIPs				
6	*C3* Arg735Trp	KASPar				
7	*C9* Cys54*	WES				
8	*CFH* 1697-17_-8delATTTTACCTT	WES, Sanger				
9	*CFH* Ala161Ser	smMIPs, Sanger	*CFH* Gln950His	smMIPs, Sanger	*C9* Met45Leu	smMIPs
10	*CFH* Gln400Lys	smMIPs, Sanger				
11	*CFH* Gly255Glu	smMIPs, Sanger				
12	*CFH* Ile184Leufs*33	smMIPs, Sanger				
13	*CFH* Gln950His	WES, exome chip				
14	*CFI* Gly188Ala	smMIPs, WES				
15	*CFH* Arg1078Ser	WES				
16	*CFH* Arg166Trp	smMIPs, WES				
17	*CFH* Trp858Arg	smMIPs, WES				
18	*C3* Lys155Gln	smMIPs	*CFH* Gln950His	smMIPs	*C9* Pro167Ser	smMIPs
19	*C9* Val69Ile	smMIPs				
20	*CFH* Ala425Val	smMIPs				
21	*CFH* Tyr243His	smMIPs	*CFB* Lys533Arg	smMIPs		
22	*CFH* Tyr916*	smMIPs, Sanger				
23	*CFI* Pro50Ala	smMIPs, Sanger				
24	*CFH* Cys357Ser	smMIPs, Sanger				
25	*CFH* Gln376*	smMIPs, Sanger				
26	*C9* Pro167Ser	smMIPs				
27	*CFH* Gly255Glu	smMIPs, Sanger				
28	*CFH* Lys204Thrfs*26	smMIPs, Sanger				
29	*CFH* Arg175Gln	smMIPs, KASPar				
30	*CFH* Arg303Gln	smMIPs, WES	*CFH* Ser193Leu	smMIPs, WES		
31	*CFH* Leu3Val	WES, smMIPs, Sanger				

© 2021 de Breuk A et al. *JAMA Ophthalmology.*

No.	Variant 1	Source variant 1	Variant 2	Source variant 2	Variant 3	Source variant 3
32	*CFH* Arg303Gln	WES, exome chip	*CFH* Ser193Leu	WES, KASPar		
33	*C3* Ser1619Arg	smMIPs				
34	*CFH* Gln950His	smMIPs, WES	*C3* Arg735Trp	smMIPs, WES		
35	*CFH* Gln846Arg	smMIPs, WES, Sanger				

Table detailing the 35 patients with early-onset drusen maculopathy carrying one or more rare variants in the complement genes *CFH, CFI, CFB, C3* or *C9*, and the corresponding genotype platform where the variant was identified. *CFH* = complement factor H, *CFI* = complement factor I, *CFB* = complement factor B, *C3* = complement 3, *C9* = complement 9, WES = whole exome sequencing, smMIPs = single molecule molecular inversion probes; empty cells indicate no additional variants were identified.

© 2021 de Breuk A et al. *JAMA Ophthalmology.*

eTable 5. Logistic regression analysis for the association of *CFH* rare variant carriership with EODM phenotype

Variable	OR	95% CI	P value
CFH rare variant carriership			
No	1 [reference]	-	-
Yes	7.209	2.657-19.562	<.001
Sex			
Male	1 [reference]	-	-
Female	2.342	1.163-4.717	.02
Smoking status			
Never	1 [reference]	-	-
Past	0.376	0.175-0.809	.01
Current	0.734	0.282-1.909	.53

Table detailing the results of the binary logistic regression analysis for the association of *CFH* rare variant carriership with EODM phenotype, adjusted for sex and smoking status. CFH = complement factor H, OR = odds ratio, CI = confidence interval.

© 2021 de Breuk A et al. *JAMA Ophthalmology.*

eTable 6. Characteristics complete and incomplete data

	Complete data		Incomplete data	
	**EODM** (n = 89 [178 eyes])	**AMD** (n = 91 [182 eyes])	**EODM** (n = 81 [162 eyes])	**AMD** (n = 82 [164 eyes])
**Sex, No. (%)**				
Male	31 (34.8)	46 (50.5)	26 (32.1)	42 (51.2)
Female	58 (65.2)	45 (49.5)	55 (67.9)	40 (48.8)
***CFH* rare variant carriership, No. (%)**	27 (30.3)	7 (7.7)	26 (32.1)	6 (7.3)
**Predominant drusen type in grid, No. of eyes (%)**				
Hard drusen	1 (0.6)	4 (2.2)	1 (0.6)	4 (2.4)
Soft drusen < C1	10 (5.6)	23 (12.6)	10 (6.2)	22 (13.4)
Soft distinct drusen	8 (4.5)	29 (15.9)	5 (3.1)	28 (17.1)
Soft indistinct drusen	154 (86.5)	75 (41.2)	141 (87.0)	64 (39.0)
Reticular drusen	3 (1.7)	27 (14.8)	3 (1.9)	22 (13.4)
Cannot grade or not applicable	2 (1.1)	24 (13.2)	2 (1.2)	24 (14.6)
**Largest drusen size within grid, No. of eyes (%)**				
< C0 (63 µm)	1 (0.6)	5 (2.7)	1 (0.6)	5 (3.0)
< C1 (125 µm)	23 (12.9)	24 (13.2)	23 (14.2)	22 (13.4)
< C2 (250 µm)	49 (27.5)	48 (26.4)	42 (25.9)	41 (25.0)
≥ C2 (250 µm)	98 (55.1)	53 (29.1)	90 (55.6)	49 (29.9)
Reticular	4 (2.2)	26 (14.3)	4 (2.5)	21 (12.8)
Cannot grade or not applicable	3 (1.7)	26 (14.3)	2 (1.2)	26 (15.9)
**Small drusen in grid, No. of eyes (%)**				
Absent	53 (29.8)	54 (29.7)	52 (32.1)	50 (30.5)
Present	121 (68.0)	104 (57.1)	107 (66.0)	90 (54.9)
Cannot grade or not applicable	4 (2.2)	24 (13.2)	3 (1.9)	24 (14.6)
**Intermediate drusen in grid, No. of eyes (%)**				
Absent	9 (5.1)	13 (7.1)	9 (5.6)	13 (7.9)
Present	165 (92.7)	145 (79.7)	150 (92.6)	127 (77.4)
Cannot grade or not applicable	4 (2.2)	24 (13.2)	3 (1.9)	24 (14.6)
**Large drusen in grid, No. of eyes (%)**				
Absent	24 (13.5)	34 (18.7)	24 (14.8)	32 (19.5)
Present	151 (84.8)	124 (68.1)	136 (84.0)	108 (65.9)
Cannot grade or not applicable	3 (1.7)	24 (13.2)	2 (1.2)	24 (14.6)
**Proportion of grid area covered by drusen, No. of eyes (%)**				
0 to 10 %	45 (25.3)	71 (39.0)	42 (25.9)	66 (40.2)
10 to 50 %	103 (57.9)	75 (41.2)	94 (58.0)	66 (40.2)
> 50 %	27 (15.2)	13 (7.1)	24 (14.8)	9 (5.5)
Cannot grade or not applicable	3 (1.7)	23 (12.6)	2 (1.2)	23 (14.0)
**Drusen outside grid, No. of eyes (%)**				
Absent	23 (12.9)	42 (23.1)	22 (13.6)	41 (25.0)
Present	153 (86.0)	139 (76.4)	138 (85.2)	122 (74.4)
Cannot grade or not applicable	2 (1.1)	1 (0.5)	2 (1.2)	1 (0.6)
**Increased pigment, No. of eyes (%)**				
Absent	75 (42.1)	75 (41.2)	69 (42.6)	67 (40.9)
< C2 (250 µm)	24 (13.5)	24 (13.2)	24 (14.8)	20 (12.2)
≥ C2 (250 µm)	77 (43.3)	81 (44.5)	67 (41.4)	75 (45.7)
Cannot grade or not applicable	2 (1.1)	2 (1.1)	2 (1.2)	2 (1.2)

© 2021 de Breuk A et al. *JAMA Ophthalmology.*

	Complete data		Incomplete data	
	**EODM** (n = 89 [178 eyes])	**AMD** (n = 91 [182 eyes])	**EODM** (n = 81 [162 eyes])	**AMD** (n = 82 [164 eyes])
**RPE degeneration, No. of eyes (%)**				
Absent	112 (62.9)	142 (78.0)	101 (62.3)	130 (79.3)
< C2 (250 µm)	15 (8.4)	10 (5.5)	14 (8.6)	8 (4.9)
< Central grid	36 (20.2)	22 (12.1)	32 (19.8)	21 (12.8)
≥ Central gid	3 (1.7)	6 (3.3)	3 (1.9)	3 (1.8)
Cannot grade or not applicable	12 (6.7)	2 (1.1)	12 (7.4)	2 (1.2)
**Geographic atrophy, No. of eyes (%)**				
Absent	141 (79.2)	109 (59.9)	126 (77.8)	97 (59.1)
Present	36 (20.2)	72 (39.6)	35 (21.6)	66 (40.2)
Cannot grade or not applicable	1 (0.6)	1 (0.5)	1 (0.6)	1 (0.6)
**Choroidal neovascularization, No. of eyes (%)**				
Absent	132 (74.2)	119 (65.4)	122 (75.3)	107 (65.2)
Present	41 (23.0)	62 (34.1)	35 (21.6)	56 (34.1)
Cannot grade or not applicable	5 (2.8)	1 (0.5)	5 (3.1)	1 (0.6)
**Serous detachment (if CNV) , No. of eyes (%)**				
Absent	11 (26.8)	16 (25.8)	11 (31.4)	16 (28.6)
Present	24 (58.5)	45 (72.6)	21 (60.0)	39 (69.6)
Cannot grade or not applicable	6 (14.6)	1 (1.6)	3 (8.6)	1 (1.8)
**Subretinal hemorrhage (if CNV) , No. of eyes (%)**				
Absent	35 (85.4)	44 (71.0)	31 (88.6)	40 (71.4)
Present	2 (4.9)	16 (25.8)	2 (5.7)	14 (25.0)
Cannot grade or not applicable	4 (9.8)	2 (3.2)	2 (5.7)	2 (3.6)
**Fibrous scar (if CNV) , No. of eyes (%)**				
Absent	13 (31.7)	12 (19.4)	9 (25.7)	9 (16.1)
Present	26 (63.4)	49 (79.0)	25 (71.4)	46 (82.1)
Cannot grade or not applicable	2 (4.9)	1 (1.6)	1 (2.9)	1 (1.8)
**Hard exsudates (if CNV) , No. of eyes (%)**				
Absent	33 (80.5)	39 (62.9)	29 (82.9)	35 (62.5)
Present	4 (9.8)	23 (37.1)	4 (11.4)	21 (37.5)
Cannot grade or not applicable	4 (9.8)	0 (0.0)	2 (5.7)	0 (0.0)

Table detailing characteristics in the complete and incomplete dataset due to missing smoking status in 17 patients. Chi-square tests and t-tests were performed between a dummy variable for smoking (0 = missing; 1 = observed) and other variables in the dataset (category, sex, GRS, disease stage, *CFH* rare variant carriership). Smoking status was missing at random, as missingness on this variable was not related to the values of the other variables in the dataset (category, sex, GRS, disease stage, *CFH* rare variant carriership). Multiple imputation was not performed. AMD = age-related macular degeneration, EODM = early-onset drusen maculopathy, CNV = choroidal neovascularization, CFH = complement factor H.

© 2021 de Breuk A et al. *JAMA Ophthalmology.*

eTable 7. Details of rare complement variants identified in patients with early-onset drusen maculopathy

Chr	Position	Gene	Nucleotide change	Protein change	PhyloP	CADD_Phred	Grantham Score	Functional effect
Chr 1	196621254	*CFH*	7C>G	Leu3Val	0.004	11.9	32	Unknown
Chr 1	196643098	*CFH*	350+6T>G	-	2.744	20.4	0	Noncoding splice site variant, predicted to severely affect splicing[7]
Chr 1	196646659	*CFH*	481G>T	Ala161Ser	-0.839	12.1	99	Normal FH levels[8]
Chr 1	196646674	*CFH*	496C>T	Arg166Trp	-0.047	22.2	101	Unknown
Chr 1	196646702	*CFH*	524G>A	Arg175Gln	-0.059	0.019	43	Reduced ability to degrade C3b[9]
Chr 1	196646728	*CFH*	550delA	Ile184Leufs*33	-100	15.04	0	*Frameshift variant*
Chr 1	196646756	*CFH*	578C>T	Ser193Leu	1.024	23.1	145	Reduced ability to degrade C3b[9]
Chr 1	196646782	*CFH*	607_610dup	Lys204Thrfs*26	0.193	33	1000	*Frameshift variant*
Chr 1	196648860	*CFH*	727T>C	Tyr243His	0.141	11.72	83	Unknown
Chr 1	196648897	*CFH*	764G>A	Gly255Glu	3.912	23.9	98	Unknown
Chr 1	196654304	*CFH*	901delG	Ala301Glnfs*22	-100	11.31	0	*Frameshift variant*
Chr 1	196654311	*CFH*	908G>A	Arg303Gln	-1.875	0.229	43	Normal FH, FI and C3 plasma levels[10]
Chr 1	196658654	*CFH*	1069T>A	Cys357Ser	3.961	23.3	112	Unknown
Chr 1	196658711	*CFH*	1126C>T	Gln376*	-1.265	34	1000	*Protein-truncating variant*
Chr 1	196659231	*CFH*	1198C>A	Gln400Lys	-2.435	0.015	53	Lower FH levels[4]
Chr 1	196659255	*CFH*	1222C>T	Gln408*	0.317	34	1000	*Protein-truncating variant*
Chr 1	196659307	*CFH*	1274C>T	Ala425Val	-1.015	0.001	64	Unknown
Chr 1	196694235	*CFH*	1697-17del	-	2.065	4.57	0	Noncoding splice site variant, predicted to affect splicing[5]
Chr 1	196706077	*CFH*	2537A>G	Gln846Arg	0.403	10.67	43	Unknown
Chr 1	196706112	*CFH*	2572T>A	Trp858Arg	4.743	24.9	101	Unknown
Chr 1	196706756	*CFH*	2748C>G	Tyr916*	-0.619	35	1000	*Protein-truncating variant*
Chr 1	196709816	*CFH*	2850G>T	Gln950His	-0.273	14.41	24	Normal FH levels, normal cofactor binding for FI[11]
Chr 1	196712682	*CFH*	3234G>T	Arg1078Ser	-6.447	1.728	110	Unknown
Chr 4	110687890	*CFI*	148C>G	Pro50Ala	5.75	24.8	27	Reduced FI levels[12]
Chr 4	110682768	*CFI*	563G>C	Gly188Ala	5.386	24.3	60	Reduced FI levels; reduced ability to degrade C3b[13]
Chr 5	39342243	*C9*	133A>T	Met45Leu	0.44	15.22	15	Elevated C9 levels, but normal TCC levels[14]
Chr 5	39342214	*C9*	162C>A	Cys54*	1.273	35	1000	*Protein-truncating variant*
Chr 5	39341781	*C9*	205G>A	Val69Ile	0.028	11.24	29	Unknown
Chr 5	39331894	*C9*	499C>T	Pro167Ser	3.414	23.9	74	Elevated C9 levels[14]

© 2021 de Breuk A et al. *JAMA Ophthalmology.*

Chr	Position	Gene	Nucleotide change	Protein change	PhyloP	CADD_Phred	Grantham Score	Functional effect
Chr 6	31918154	*CFB*	1598A>G	Lys533Arg	3.242	15.95	26	Unknown
Chr 19	6718146	*C3*	463A>C	Lys155Gln	-0.429	14.8	53	Decreased binding of C3b to CFH[15]
Chr 19	6707129	*C3*	2203C>T	Arg735Trp	0.721	23.1	101	Normal MCP binding, FI cofactor activity, FB and FH binding[16]
Chr 19	6678030	*C3*	4855A>C	Ser1619Arg	0.671	22.5	110	Unknown

Table detailing the annotation, prediction scores and functional effects of the identified rare complement variants. Chr = chromosome, CADD = combined annotation dependent depletion, *CFH* = complement factor H, *CFI* = complement factor I, *CFB* = complement factor B, *C3* = complement 3, *C9* = complement 9, FH = factor H, FI = factor I, FB = factor B, MCP = membrane cofactor protein

© 2021 de Breuk A et al. *JAMA Ophthalmology.*

eFigure 1. Patients with EODM with large colloid drusen and temporal drusen

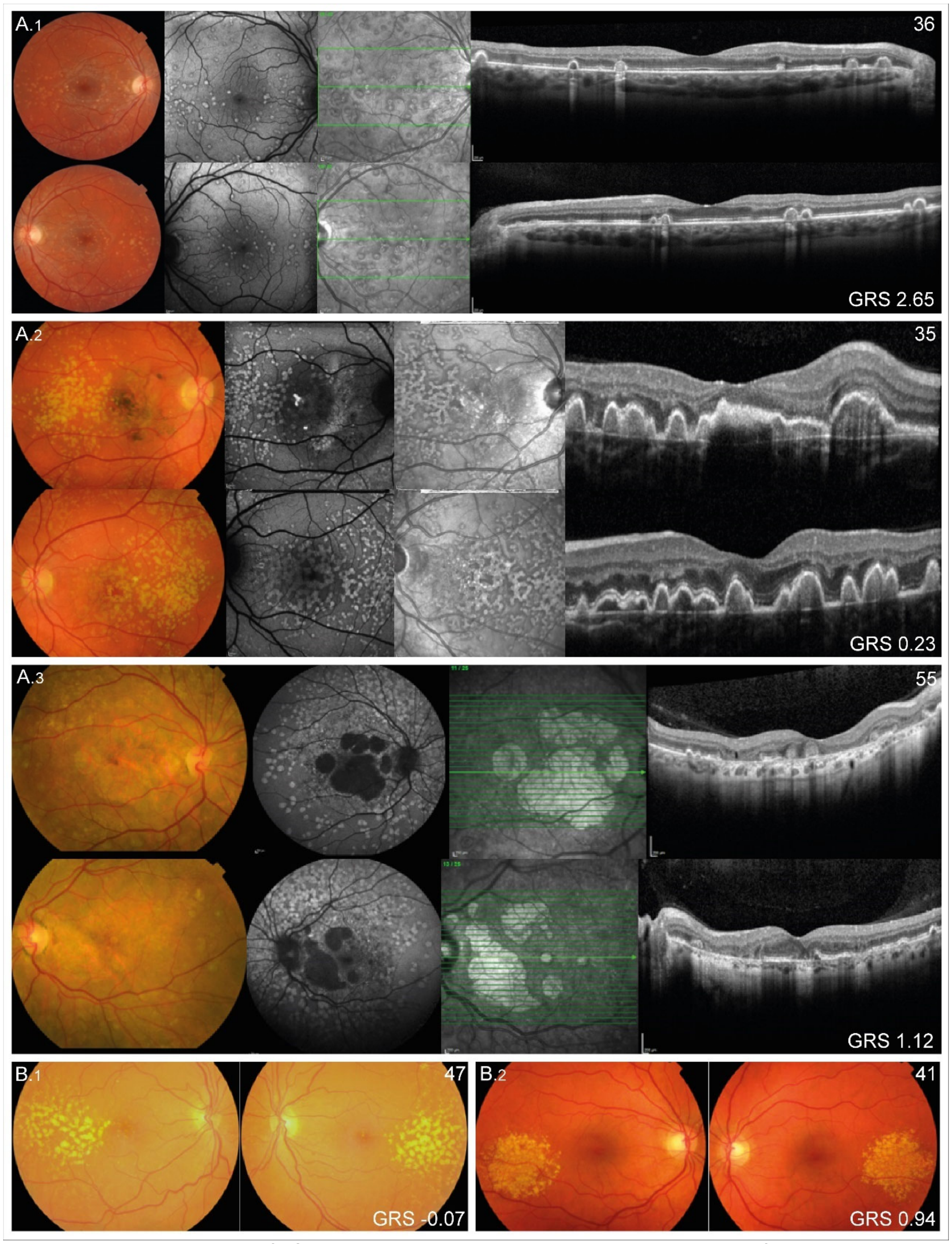

Figure detailing three examples of EODM patients with large colloid drusen and two examples of patients with drusen located temporal to the macula. Panel A: color fundus photographs of all three patients showed large yellow lesions. The location of the lesions varied from predominantly outside the vascular arcades (panel A3), to predominantly temporal to the macula (panel A2) and spread across the whole posterior pole (panel A1). Fundus autofluorescence images showed hyperautofluorescent lesions surrounded by hypoautofluorescent halos. Infrared reflectance images showed white round lesions surrounded by dark halos. Optical coherence tomography scans showed dome-shaped sub-retinal pigment epithelium deposits (panels A1 and A2; in panel A3 the central macula was dominated by atrophy). The patient in panel A2 is carrier of rare variants in the complement factor H (*CFH*) gene (Gln950His) and in the Complement 3 (*C3*) gene (Arg735Trp). Panel B1: color fundus photographs of a 47-year-old female with drusen located temporal to the fovea. Panel B2: color fundus photographs of a 41-year-old female with drusen isolated temporal to the fovea. Numbers in the right upper corner of each panel represent the age of the patient. The genetic risk score (GRS) is depicted in the right bottom corner of each panel.

© 2021 de Breuk A et al. *JAMA Ophthalmology.*

eFigure 2. Genetic risk score distribution in EODM and AMD cohorts

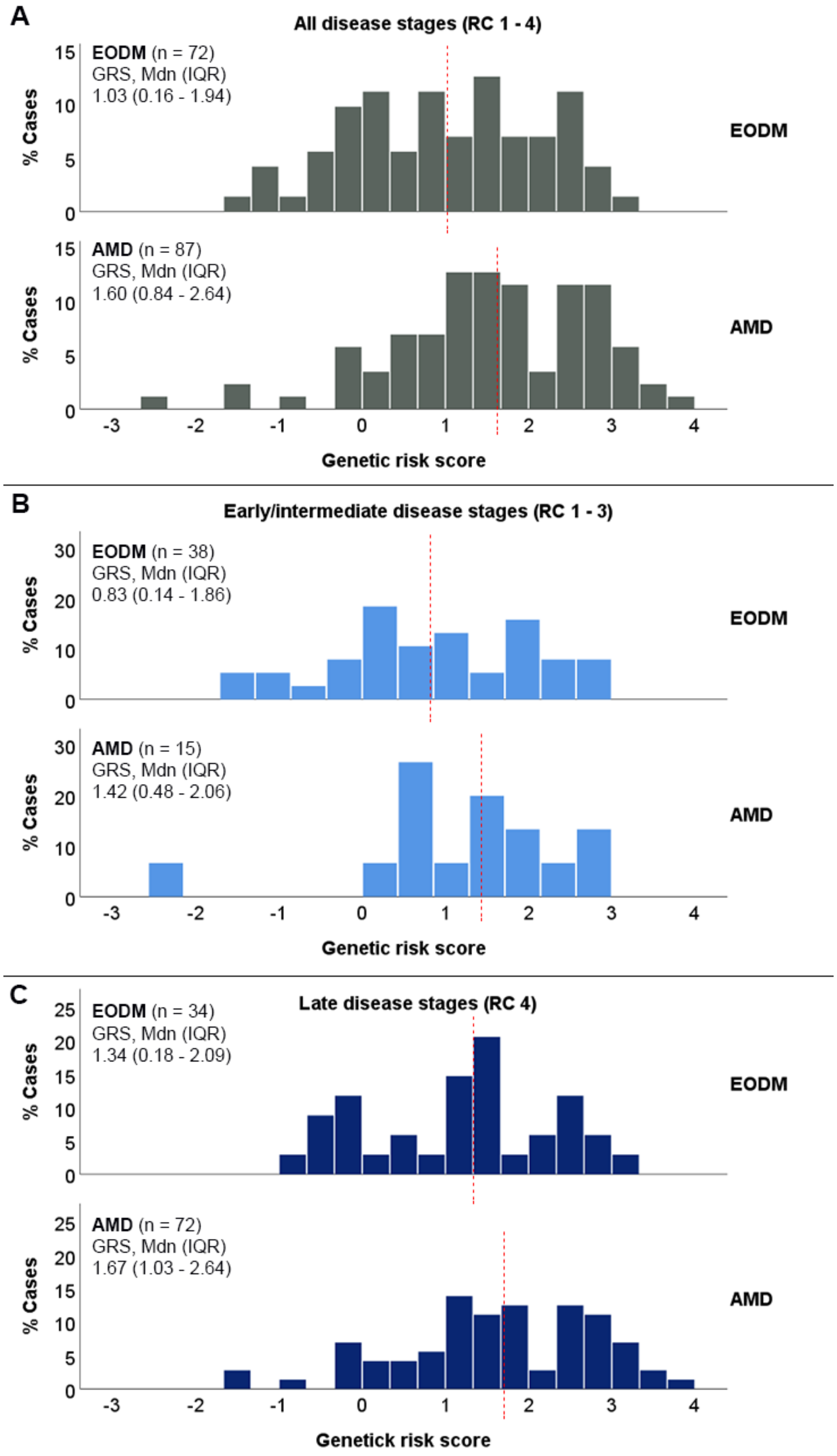

Figure detailing the distribution of the genetic risk score between patients with early onset drusen maculopathy and age-related macular degeneration. Panel A: all disease stages (RC 1-4). Panel B: only early and intermediate (RC 1-3) disease stages. Panel C: only late (RC 4) disease stages. Red dotted lines represent the median GRS. EODM = early onset drusen maculopathy, AMD = age-related macular degeneration, GRS = genetic risk score, RC = Rotterdam classification, Mdn = median, IQR = interquartile range.

© 2021 de Breuk A et al. *JAMA Ophthalmology.*

eFigure 3. Patient with *PRPH2* Arg142Trp mutation

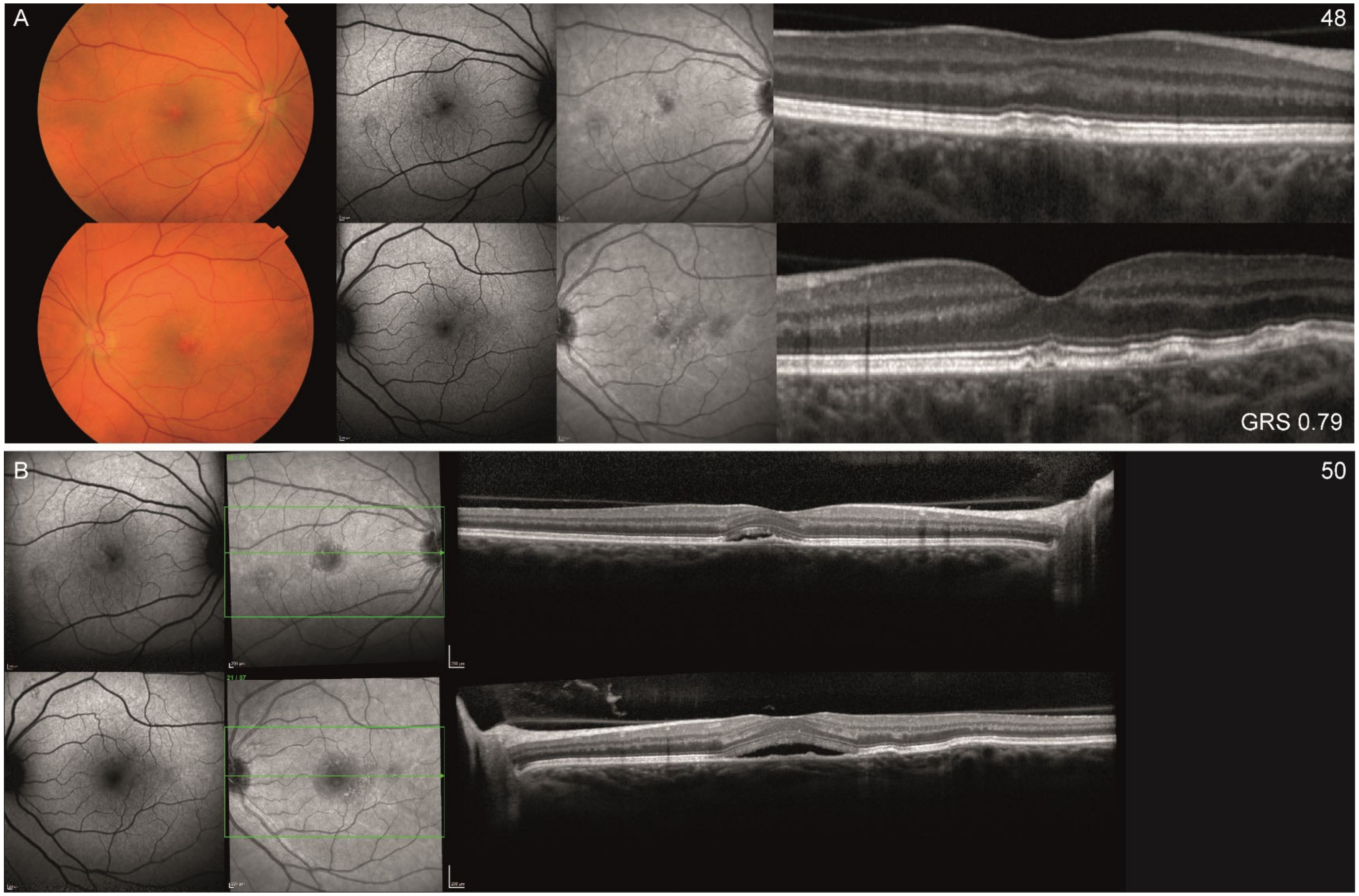

Figure detailing a 48-year-old female primarily diagnosed with early onset drusen maculopathy. Panel A: color fundus photographs showed small yellow deposits and retinal pigment epithelium alterations in the fovea. Optical coherence tomography images showing small elevations of the retinal pigment epithelium in and around the fovea. Panel B: follow-up images of the same patient after two years. Optical coherence tomography images showed a serous detachment in the fovea in both eyes. Numbers in the right upper corner of each panel indicate the age of the patient. The genetic risk score is depicted in the right bottom corner of panel A. GRS = genetic risk score.

© 2021 de Breuk A et al. *JAMA Ophthalmology.*

eReferences

1. Appel GB, Cook HT, Hageman G, et al. Membranoproliferative glomerulonephritis type II (dense deposit disease): an update. *J Am Soc Nephrol.* 2005;16(5):1392-1403.
2. Dalvin LA, Fervenza FC, Sethi S, Pulido JS. Manifestations of Complement-Mediated and Immune Complex-Mediated Membranoproliferative Glomerulonephritis: A Comparative Consecutive Series. *Ophthalmology.* 2016;123(7):1588-1594.
3. Abrera-Abeleda MA, Nishimura C, Smith JL, et al. Variations in the complement regulatory genes factor H (CFH) and factor H related 5 (CFHR5) are associated with membranoproliferative glomerulonephritis type II (dense deposit disease). *J Med Genet.* 2006;43(7):582-589.
4. Dragon-Durey MA, Fremeaux-Bacchi V, Loirat C, et al. Heterozygous and homozygous factor h deficiencies associated with hemolytic uremic syndrome or membranoproliferative glomerulonephritis: report and genetic analysis of 16 cases. *J Am Soc Nephrol.* 2004;15(3):787-795.
5. van de Ven JP, Boon CJ, Fauser S, et al. Clinical evaluation of 3 families with basal laminar drusen caused by novel mutations in the complement factor H gene. *Arch Ophthalmol.* 2012;130(8):1038-1047.
6. Han DP, Sievers S. Extensive drusen in type I membranoproliferative glomerulonephritis. *Arch Ophthalmol.* 2009;127(4):577-579.
7. Boon CJ, Klevering BJ, Hoyng CB, et al. Basal laminar drusen caused by compound heterozygous variants in the CFH gene. *Am J Hum Genet.* 2008;82(2):516-523.
8. Servais A, Noël LH, Roumenina LT, et al. Acquired and genetic complement abnormalities play a critical role in dense deposit disease and other C3 glomerulopathies. *Kidney Int.* 2012;82(4):454-464.
9. Geerlings MJ, Kremlitzka M, Bakker B, et al. The Functional Effect of Rare Variants in Complement Genes on C3b Degradation in Patients With Age-Related Macular Degeneration. *JAMA Ophthalmol.* 2017;135(1):39-46.
10. Fakhouri F, Jablonski M, Lepercq J, et al. Factor H, membrane cofactor protein, and factor I mutations in patients with hemolysis, elevated liver enzymes, and low platelet count syndrome. *Blood.* 2008;112(12):4542-4545.
11. Mohlin FC, Nilsson SC, Levart TK, et al. Functional characterization of two novel non-synonymous alterations in CD46 and a Q950H change in factor H found in atypical hemolytic uremic syndrome patients. *Mol Immunol.* 2015;65(2):367-376.
12. Kavanagh D, Yu Y, Schramm EC, et al. Rare genetic variants in the CFI gene are associated with advanced age-related macular degeneration and commonly result in reduced serum factor I levels. *Hum Mol Genet.* 2015;24(13):3861-3870.
13. van de Ven JP, Nilsson SC, Tan PL, et al. A functional variant in the CFI gene confers a high risk of age-related macular degeneration. *Nat Genet.* 2013;45(7):813-817.
14. Kremlitzka M, Geerlings MJ, de Jong S, et al. Functional analyses of rare genetic variants in complement component C9 identified in patients with age-related macular degeneration. *Hum Mol Genet.* 2018;27(15):2678-2688.
15. Seddon JM, Yu Y, Miller EC, et al. Rare variants in CFI, C3 and C9 are associated with high risk of advanced age-related macular degeneration. *Nat Genet.* 2013;45(11):1366-1370.
16. Frémeaux-Bacchi V, Miller EC, Liszewski MK, et al. Mutations in complement C3 predispose to development of atypical hemolytic uremic syndrome. *Blood.* 2008;112(13):4948-4952.

© 2021 de Breuk A et al. *JAMA Ophthalmology.*
